# The Construction of Image Reference Points and Text Appeals Information Tailoring in Promoting Diners’ Public Environment Maintenance Behavior Intention

**DOI:** 10.3390/ijerph192114477

**Published:** 2022-11-04

**Authors:** Yanfei Zhu, Yuli Wang, Ying Li, Xiaoxi Du, Qi Guo, Mo Chen, Yun Lin

**Affiliations:** 1 School of Mechanical Engineering, Southeast University, Nanjing 211189, China; 2 Department of Art and Design, Beijing University of Chemical Technology, Beijing 100029, China; 3 School of Art Design and Media, East China University of Science and Technology, Shanghai 200237, China; 4 School of Art and Design, Nanjing University of Technology, Nanjing 210031, China; 5 School of Design Art and Media, Nanjing University of Science and Technology, Nanjing 210014, China

**Keywords:** public environment maintenance, image reference points, text appeals, information tailoring, pro-environmental behavioral intention

## Abstract

The environmental maintenance of public dining spaces significantly impacts urban construction’s sustainable and healthy development. This paper studied the influence of image–text information tailoring relationships on behavioral intentions to promote public dining space environment maintenance. We used a two-factor between-subject experimental design, two (image reference points: self vs. others) × two (text appeals: feasibility vs. desirability). We also examined the mediating roles of environmental maintenance attitudes and environmental responsibility perceptions as regards pro-environmental behavioral intentions. The research results showed the following: (1) Among the four image–text information construction methods, the other’s image reference point with the desirability text appeal promotes the diners’ pro-environmental behavioral intention with optimal effectiveness; and (2) Environmental maintenance attitudes and environmental responsibility perceptions play mediating roles in promoting diners’ intention to maintain environmental behavior in the image–text combined information presentation. Environmental responsibility perceptions cannot be mediated alone and must be progressively mediated with environmental maintenance attitudes.

## 1. Introduction

The cleaning and maintenance of the public space environment are very important for green and healthy city development [1]. People are the main body operating in the public space, and especially during the global COVID-19 period, implementing public environmental sanitation measures is urgent and necessary [2]. Law and regulation control, improving practitioners’ responsibility sense, and servicing audiences’ awareness are the three main ways to alleviate public health problems [3]. However, from the social rule perspective, warnings and coercion are more important after the emergence of uncivilized problems. From the practitioner’s perspective, compared with service audiences, excessive reliance dramatically increases the labor costs and workload [4]. By improving the service audience’s awareness, and establishing national conscious and spontaneous good hygiene habits, public environmental problems can be alleviated and enhanced at the source, and public order and good customs can be created [5,6].

Diet is one of the most important components of human society, and a fast-paced work and lifestyle have significantly increased people’s likelihood of eating out [7]. Uncivilized dining behaviors bring related public health problems, such as hospitalization and even death caused by food-borne disease outbreaks [8,9], and also generate social operating costs and burdens in public dining environments. In fact, the cleanliness of the public dining environment also indirectly determines diners’ consumer satisfaction and return intention [10]. The World Health Organization issued guidance on public health measures in the context of COVID-19 in May 2020 [11]. Many countries worldwide have taken the lead in the public environment to improve satisfaction and tangible service quality [12]. Based on the overall decrease in satisfaction with the public cleanliness of hawker centers in 2021, the Singapore Environment Agency launched slogans such as the “Clean Table Campaign” in 2022, trying to change the uncivilized environmental maintenance behaviors in public spaces by controlling social rules and improving the service audience’s awareness levels [13]. Based on the eating habits of sitting around tables, China in 2020 launched the “Proposal to Promote Public Chopsticks and Spoons to Build a Civilized Dining Table”, and released a series of publicity materials to promote and encourage healthy public dining behaviors [14].

Information communication is an effective means of awareness training, providing potential conditions for improving public health [15,16]. How to effectively construct and disseminate information widely, and cultivate the audience’s positive attitude and behavioral intention, is of great significance in creating a sustainable lifestyle and healthy social development [17,18,19]. In July 2021, Japan launched a survey on the acceptance of green environment maintenance behaviors in 12 countries and regions worldwide. The results show that in information dissemination, the United States, Japan, and Europe mainly emphasize “self”. In contrast, China and the ASEAN region seemed to feel that the “public” can have a stronger behavioral perception effect on the masses [20]. That is to say, when the information matches the audience’s ideology, it can achieve better behavioral intentional influences. Correspondingly, in the construction of public environment maintenance information, whether the inclusion of reference points can regulate or strengthen the information itself and the amplification effect on users’ pro-environmental behavioral intentions is an important question in this study.

In this study, we conducted a two-factor online experiment—two (image reference points: self vs. others image reference point) × two (text appeals: feasibility vs. desirability text appeals)—to explore the effect of image–text combination information tailoring the construction on the effectiveness of diners’ maintenance perception and behavior intention in public dining environments.

## 2. Literature Review

### 2.1. Feasibility and Desirability Appeals in Information Tailoring

The construal level theory was first proposed by Trope and Liberman, and it proposes that users’ responses to information depend on mental representations. The mental representation of information differs as regards the abstraction level, that is, the construal level [21]. High-construal level representations are relatively abstract, and mainly characterize the subject and meaning of information. On the other hand, low–high construal level representations are rather concrete, mainly representing the details and specificity of the information [22]. Based on this theory, two content levels are often used to distinguish abstract representations from concrete representations in information construction and communication, namely, desirability appeals and feasibility appeals [23]. In goal-oriented activities, desirability usually represents a high-level explanation, while feasibility usually represents a low-level explanation [24].

Reczek and Trudel have shown through experiments that construal information at the abstract (vs. concrete) level is associated with more positive responses to environmentally friendly products. Different responses are driven by information construal and attention to temporal distance [25]. Han et al. explored how feasibility, desirability, and anthropomorphism shape environmental persuasion in the context of a sustainable recycling advertisement, showing that feasibility is more effective than desirability [26]. Sevincer and Oettingen, based on the theory of alcohol myopia, found that students who choose to drink alcohol are more influenced by preference than feasibility in their personal goal selection, compared to taking a placebo [27]. Tok et al.’s research on product advertising showed that when far-end spatial distance and desirability match, and near-end spatial distance and feasibility match, it can lead to more favorable product evaluations [28]. Ding et al. explored the impact of abstract and concrete interpretations of air pollution visibility on the way consumers process information, as well as users’ preference for desirability in product trade-offs [29]. Yoon et al. studied consumers’ demands for speed in the presentation of marketing communication advertisements. They showed that slow-moving advertisements elicit high construal levels, elevating the desirability of the product for consumers. Conversely, fast-moving advertisements elicit low construal levels, causing the feasibility of the advert’s claims about the product’s attributes to be emphasized for the consumer [30]. Kim et al. examine how the Facebook page set-up affects the information validity of different construal levels. Individuals who saw news summary pages responded more positively to desirability-focused messages of abstract frames. In contrast, individuals who saw timeline pages responded more positively to desirability-focused messages with a concrete framework [31]. Tugrul and Lee discussed how to improve the communication persuasion of donation activities on social media. The study shows that the gain frame and desirability matching, and the loss frame and feasibility matching, are more effective in persuading donation willingness [32]. Kazakova et al. explored viewers’ cognitive attitude responses to media multitasking, and the moderating effect of attractiveness. The findings suggest that media multitasking can positively impact attitudinal responses to TV advertisements, but only for those that emphasize product desirability [33]. Su et al. explored the mechanism of users sharing tourism experience information. The experimental results showed that challenging tourism activities will lead to more desirable sharing, and relaxing tourism activities will lead to more feasible sharing. Eudaimonia (hedonic) mediates tourism happiness perception [34]. Baskin et al. proposed a giver and receiver trade-off between desirability and feasibility based on a sociological perspective. The giver is more sensitive to social distance than the receiver, such that they will explain the gift more abstractly [35]. Jia et al. explored the asymmetric impact of regulatory focus on the expected desirability and desirability of using self-service technology (SST) in retail settings. Promotional focus helps consumers to recognize the desirable consumption value and feasibility of using SST, whereas prevention focus hinders consumers from understanding the feasibility-related attributes of SST [36].

Based on the relevant experimental research performed on the construal level, it can be seen that in information construction, feasibility and desirability have strong effects. The user’s background differences, information content, visual presentation methods, and other factors can cause them to process information differently, leading to differences in perception and performance asymmetry in behavioral intentions. Whether this can play a role in influencing user perception, attitude, and behavioral intervention is closely related to the match between the combined variables.

### 2.2. Reference Points in Information Tailoring

The psychological distance effect refers to the user’s subjective perceptual experience of events or objects, which are mainly divided into temporal distance, spatial distance, social distance, and hypothetical [37]. Among them, social distance refers to using oneself as a reference point to judge the difference between social objects and the self [1]. The “reference point effect” was first proposed by Kahneman and Tversky. It is not based on the possible resulting absolute utility value of the decision-making scheme but is based on an existing reference point. The decision result is the deviation direction, and the degree of the actual profit and loss from the psychological point of view [38].

Today, the reference point effect has been widely used in the construction of information tailoring in various fields. Zhang et al. demonstrated through online advertising that regulatory focus and reference points in the information framework interact with green appeal persuasion, and that perceived intrinsic motivation and attitude serially mediate the effects on behavior [39]. Through experimental research, Segev et al. determined the matching relationship between the reference point and message frame. The loss frame and the self-reference point combination can lead to a better green advertising response [40]. Chen et al. examined the perceived importance, attitudes, and behavioral intentions related to food waste in dining establishments through an information focus and message frame. Research shows that information focus can have an impact on attitudes and behaviors [41]. Fu et al. demonstrated through an eye-tracking experiment that the reference point and the message frame have an interactive effect on the visual cognition of advertisements for the use of recycled water in green communities, and the reference point has a moderating impact on the message frame [42]. Lu et al. examined the effectiveness of consistently matching reference points and the message frame in intervening in risk perception and behavioral intention construction in human–black bear conflicts [43]. In an online experiment, Nab et al. explored the effects of information framing and content reference points on homeowners’ environmental and behavioral intentions in relation to climate change. The results show that, when negative frames and self-reference points match, they can significantly influence behavioral intentions [44]. Lorenz explored the matching relationship between reference points and message frames in prosocial persuasion appeals [45]. Guo and Cao developed strategies by updating the reference point, which increased the willingness of individuals to receive the vaccine, and effectively improved vaccination coverage [46]. Karle and Schumacher explored the loss aversion theory, changing the consumers’ reference point to achieve the best matching value of products and individuals, and formulated improved strategies for advertising in the market [47]. Hu et al. found that changes of the label reference point’s location may better convey the food’s relevant attributes, thereby affecting consumers’ evaluations of genetically modified food [48]. Ruth and York explored how consistent or inconsistent relationships between information sources, reference points, and information types can impact stakeholders’ attitudes towards a firm’s reputation [49].

Through relevant research, it can be seen that reference point setting in information construction plays a vital role in users’ attitudes and intentions. In particular, the design of information content that matches the reference point can have a significantly higher impact on attitude and behavior than non-matching information content design and has a corresponding value effect.

### 2.3. Construction of Feasibility, Desirability and Reference Points in Information Tailoring

The four dimensions of psychological distance have the characteristics of interrelatedness and mutual influence. When they are consistent, they will strengthen the user’s perception and behavioral stimuli [50,51,52,53]. At the same time, the two-way relationship between psychological distance and construal level is also interconnected, automatically activated, and consistent, and does not require conscious participation. Users often use high construal levels to represent things further away, and low construal levels to represent things closer in terms of psychological distance [54]. Bar-Ana et al. verified the association between construal level-related words (low and high) and four psychological distances (close and far) through an implicit association test and explored whether the construal level and psychological distance of word concepts being consistent would influence users’ perception and judgment [55]. Based on social psychology experiments, Lu et al. showed that users pay more attention to feasibility than desirability in decision making for themselves and others [56]. Hsieh and Yalch studied how maximizers and satisfiers drive preferences for desirable and feasible attributes, and influence product choices when seeing product advertisements [57]. Lee et al. explored the matching relationship between users with different political beliefs and the tailoring of preferences related to feasibility and desirability in anti-tobacco information [58].

Many experiments have confirmed that the user’s construal level will change with changes in psychological distance to the event, thus affecting the response and judgment. The improvement of effectiveness brought about by the pursuit of matching in information construction has become ubiquitous in construction methods. Further, we query whether there is a matching effect in the construction of construal level appeals (feasibility and desirability) and the social distance (other-reference point and self-reference point), and how the effective combination affects users’ attitudes and behavioral intentions in the maintenance of the public environment. In addition, as far as we know, sociology and communication studies have so far mainly focused on textual research, and there are relatively few studies on the relationship between image and text in information matching construction. Therefore, we propose the following hypotheses based on the above theories and research gaps.

**Hypothesis 1**. *Text appeals and reference point have significant interaction effects on the attitudes, responsibility, and behavioral intentions of public environment maintenance*.

**Hypothesis 1 (H1a)**. *When the self-reference point and feasibility information are constructed, it significantly impacts the attitude, responsibility, and behavioral intention of public environment maintenance*.

**Hypothesis 1 (H1b)**. *When the other-reference point and desirability information are constructed, it significantly impacts the attitude, responsibility, and behavioral intention of public environment maintenance*.

### 2.4. The Mediating Role of Perceived Environmental Responsibility in Pro-Environmental Behavior Intention

Pro-environmental behavioral intention is an important mediator of pro-environmental behavior [59]. The factors affecting pro-environmental behavior can be divided into exogenous situational factors and endogenous psychological factors [60]. Yoon et al. emphasized that social responsibility plays a crucial mediating role in the relationship between risk perception and pro-environmental behavioral intentions, suggesting that public eco-friendly behaviors are encouraged in a way that motivates individuals to take responsibility for the environment [61]. Cuadrado et al. analyzes how conscientiousness interacts with an implicit theory about climate change (ITCC) to mediate pro-environmental intentions. The results show that ITCC plays a moderating role between conscientiousness and pro-environmental behavioral intentions [62]. Chang et al. examined the moderating role of environmental motivation and environmental knowledge between information framing and pro-environmental behavioral intentions through a questionnaire survey [63]. Truong et al. investigated the relationship between customer perception, satisfaction, and behavioral intention in Vietnamese restaurants, and the results show that customer perception affects satisfaction and behavioral intention [64]. Kumar et al. used the theory of planned behavior (TPB) to determine that combining environmental concerns, personal ethics, and perceived validity properties predict Indian youth’s purchase intentions for environmentally friendly clothing. Among them, perceived consumer effectiveness has a moderating effect on attitude intention [65]. Kautish et al. established a theoretical model to explore the structure of the moderation of green purchasing behavior (GPB) by environmental awareness and recycling intention [66]. Zheng et al. used the TPB and CAB models to develop a conceptual model of TPEBI, which explored the impact on TPEBI of the multiple dimensions of attitude, subjective norm, perceived behavioral control, unique destination charm, and tourist pleasure [67]. Pivetii et al. conducted structural equation modeling on the recycling intention of municipal solid waste in southern Italy. The results show that internal attribution and social norms were the strongest predictors of attitudes, and attitudes strongly predicted behavioral intentions, which predicted pro-environmental behaviors. Attitudes mediate internal attributions, social norms, and behavioral intentions [68]. Lin et al. analyzed the moderating role of individualism and collectivism in national culture in pro-environmental behavior and provided a reference strategy for tourism and hotel marketing information formulation [69]. Through the pro-environmental behavior task (PEBT) in the Vehicle Energy Saving Experiment, Lange showed that relevant variable control affects actual pro-environmental behavior [70]. Bleidorn et al. used a large-sample survey of the Swiss population to show that pro-environmental attitudes could predict pro-environmental consumer behavior, and the life stage of the population plays a moderating role between the two [71].

Based on the above literature, it can be found that in promoting user behavior in environmental protection, attitudes have a positive mediating effect between behavioral intentions and behavior implementation. Among the influences on attitudes, perceptions at the social level are the strongest. We then queried whether, as the core of the social operation mechanism, the user’s perception of responsibility for their position and relative position has a mediating effect on information and pro-environmental behavioral intentions. Therefore, we proposed the following hypotheses:

**Hypothesis 2**. *Environmental maintenance attitudes mediate the interaction effect between text appeals and reference point on pro-environmental behavioral intentions*.

**Hypothesis 3**. *Environmental maintenance attitudes and environmental responsibility perceptions form a cascade of progressive mediating relationships on pro-environmental behavioral intentions*.

## 3. Methodology

### 3.1. Experimental Participants

Our questionnaire was distributed through the online questionnaire platform Questionnaire Star. A total of 230 subjects were recruited to participate in this online experiment. The subjects were mainly young and middle-aged, and all had a certain frequency of eating in and out in the public dining environment (121 males, 109 women). After completing the questionnaire, all subjects received a small gift as thanks for participating in the experiment. After excluding abnormal data, 228 data sets (119 males, 109 females) were collected.

### 3.2. Stimulus Material Design

**Figure 1:**
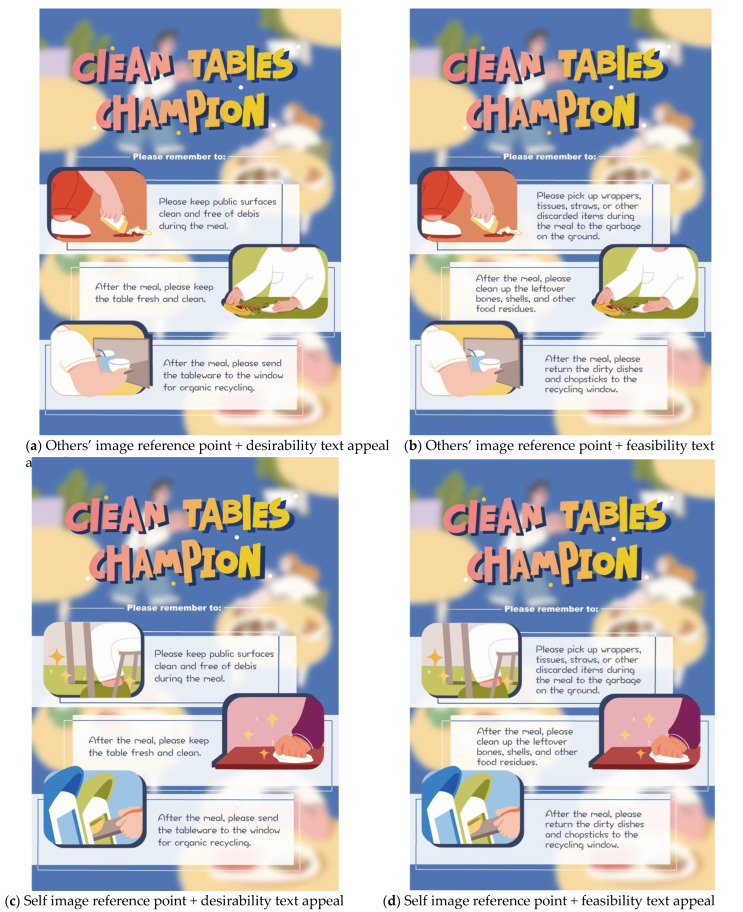
Stimulus materials design. (Note: The original four groups of presentation materials are in Chinese, and we translated them into English for this paper).

Our experimental stimuli use a combination of two levels of image reference points (self vs. others) and two levels of text appeals (feasibility vs. desirability) to generate four static public dining space environmental maintenance advertising messages (see Figure 1). To maintain the content elements of the public environment, we selected three typical behaviors during and after dining out: (a) cleaning up food residues on the table; (b) dropping meal-related garbage; and (c) returning tableware and residual garbage after use. The image reference points used are “self” and “others”, in the context of social psychological distance, and these were used to generate behaviors from the “first perspective” and “third perspective” as the two visual perspectives of content presentation in the public dining space [72,73]. The textual appeals mainly use “feasibility” and “desirability” as the two types of construal level to display content from a semantic perspective [26,74].

### 3.3. Experimental Measures

Manipulation checks: to examine the information content presentation, we recruited 10 subjects to test whether our combined information design at the reference point and text appeal construal levels were successful, using a 7-point Likert Scale. The t-test results show that the self-reference point was closer in psychological distance than the others-reference point (M_self_ = 6.1, SD = 0.876; M_others_ = 3.9, SD = 1.1, t = 4.947, *p* < 0.05). Compared with the text desirability appeal, the text feasibility appeal was greater at the construal level (M_desirability_ = 5.8, SD = 0.789; M_feasibility_ = 3.9, SD = 1.197, t = 4.191, *p* < 0.05). Therefore, our horizontal information manipulation was successful.

Self-reported behavioral frequency measures: we used Truelove’s environmental self-reported behavior frequency to assess past behavior [75]. The past behavior item was “Please state your best estimate of how often you eat out: ‘Once every three months’, ‘One or more times per month’, ‘Once a week’, ‘Multiple times per week’, and ‘One or more times per day’”.

Environmental maintenance attitude: we based Lee’s [76] environmental attitude scale-adapted environmental maintenance attitude scale. The three items were “Environmental maintenance is critical to the sustainable development of public restaurants”; “I strongly agree that public restaurants need environmental maintenance”; “Environmental awareness in public restaurants is critical among diners”. A 7-point Likert scale (1 = strongly disagree; 7 = strongly agree) indicates the subjects’ level of agreement with these items. The internal consistency of the scale was α = 0.930.

Environmental responsibility perception: we used Lee’s [76] three statements—“Supporting the environment makes me feel like I am an environmentally responsible person”; “I am proud to be an environmentalist”; “Supporting the environment makes me feel meaningful”—to assess environmental responsibility perceptions. We used the average of three 5-point Likert scales (1 = strongly disagree; 5 = strongly agree) to assess environmental maintenance perceptions. The internal consistency of the scale was α = 0.923.

Pro-environmental behavior intention: we based Grazzini’s [77] environmental recycling intention scale and Truelove’s [75] environmental intention measures adapted pro-environmental behavior intention scale. The three items were “How likely are you to implement environmental maintenance behaviors in public restaurants?”; “How prone are you to maintaining the public restaurant environment?”; “Are you willing to implement environmental maintenance behaviors in public restaurants?” A 9-point Likert scale (1 = extremely unlikely to do this; 9 = extremely likely to do so) was measured to quantify. The internal consistency of the scale was α = 0.938.

Demoimage data: we also referred to the relevant demoimage data set out in Changand Wu [63] to collect potential variables that may affect pro-environmental behavior, such as age, gender, and education level.

### 3.4. Experimental Procedures

This study mainly examined the interactive effects of the two independent variables of image reference points’ and text construal levels’ feasibility and desirability on pro-environmental behaviors. The Likert scale was used to investigate three dependent variables: the frequency of behaviors and environmental maintenance attitudes, environmental responsibility, and subjective measurements. In addition, we also used the PROCESS SPSS macro to examine the mediating role of environmental maintenance attitudes and environmental responsibility perceptions in pro-environmental behaviors.

We recruited 230 subjects to participate in the questionnaire. After reading the participants’ informed consent forms, they were randomly assigned to one of four groups. First, they were asked to answer (1) demoimage questions; and (2) self-reported behavioral frequency questions. Secondly, the participants were asked to examine the corresponding group’s “Clean Tables Champion” E-poster, and give answers related to: (3) environmental maintenance attitude; (4) environmental responsibility perception; and (5) pro-environmental behavior intention, for a total of five groups of questions.

## 4. Result

### 4.1. Sample Profile

A total of 230 questionnaires were distributed in this experiment. After excluding two invalid questionnaires, 228 valid questionnaires were included in the final dataset for analysis. Descriptive statistics show that 64.8% of the participants were between the ages of 18 and 40, 54% were women, and 46% were men, mostly young and middle-aged. In terms of education, 84.21% of the subjects had a college degree or above.

### 4.2. Hypothesis Testing

We used image reference points and text appeals as the independent variables. We conducted a two-way analysis of variance (ANOVA) with the three dependent variables of behavioral basis frequency and environmental maintenance attitudes, environmental responsibility, and pro-environmental behavioral intentions. “Environmental responsibility perception” and “Pro-environmental behavior intention” are transformed into a 7-point Likert scale perform data analysis.

The results showed that, in the behavioral basis frequency survey, there was no significant difference in the frequency of environmental maintenance behaviors among the four groups (F(3224) = 0.058, *p* > 0.05). There was no main effect on environmental maintenance attitude (F(1224) = 0.972, *p* > 0.05), environmental responsibility perception (F(1224) = 0.061, *p* > 0.05), or pro-environmental behavioral intentions (F(1224) = 0.273, *p* > 0.05).

There was no significant main effect of text appeal on environmental maintenance attitudes (F(1224) = 0.501, *p* > 0.05), environmental responsibility perceptions (F(1224) = 0.242, *p* > 0.05), or pro-environmental behavior intentions (F(1224) = 0.5, *p* > 0.05).

Consistent with H1, image reference points and text appeals showed an interaction effect on environmental maintenance attitudes (F(1224) = 0.023, *p* < 0.05), environmental responsibility perceptions (F(1224) = 0.028, *p* < 0.05), and pro-environmental intentions (F(1224) = 0.031, *p* < 0.05) (see Table 1).

**Figure 2:**
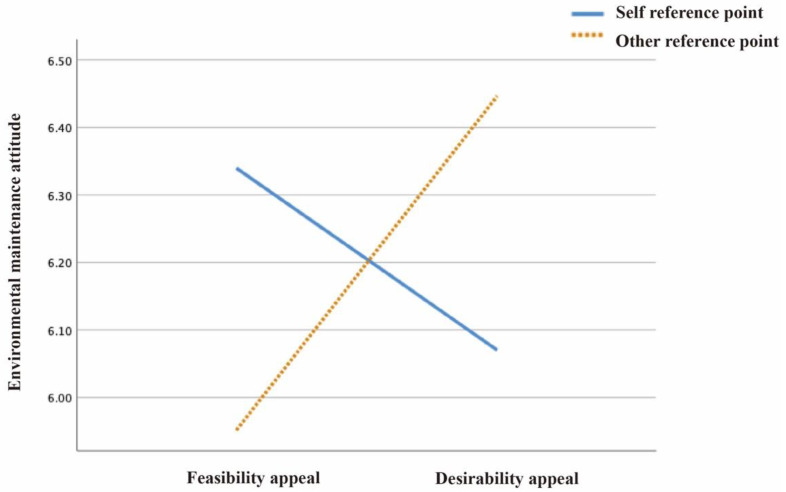
The effect of text appeals on environmental maintenance attitude, assessed by image reference point.

**Figure 3:**
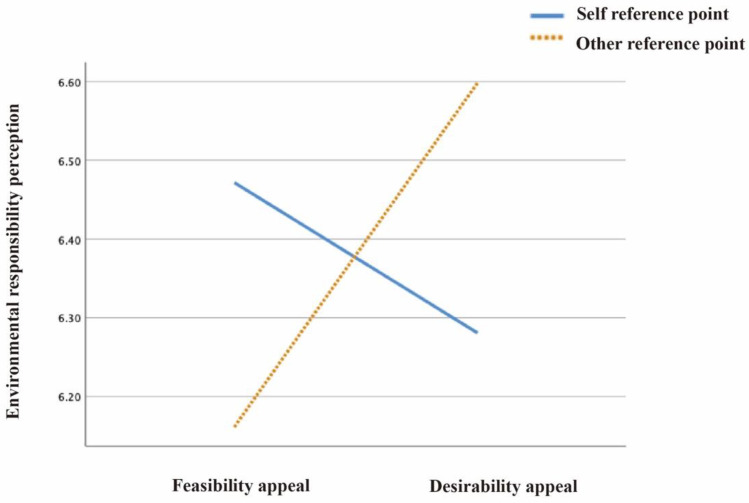
The effect of text appeals on environmental responsibility perception, assessed by image reference point.

**Figure 4:**
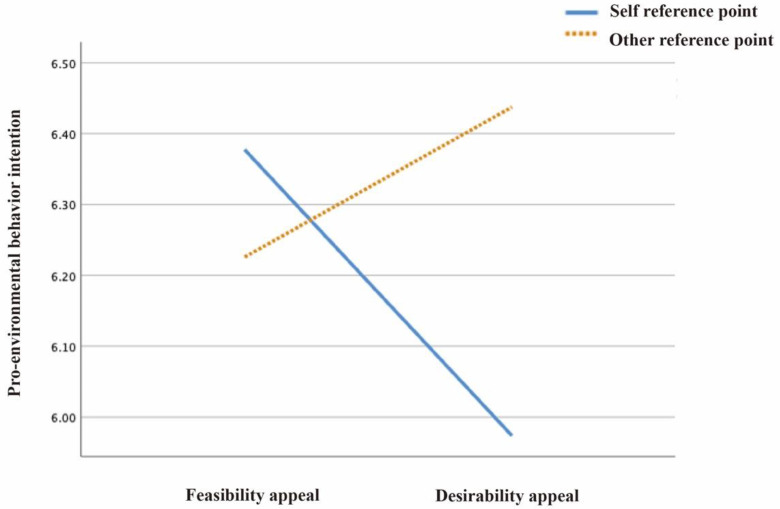
The effect of text appeals on pro-environmental behavioral intentions, assessed by image reference point.

(a) The pairwise comparison data showed that when the reference point was the self, matched with feasibility (M = 6.341, SD = 0.939), the user’s environmental maintenance attitude was stronger than when matched with desirability (M = 5.952, SD = 1.796), and there was no significant difference between the two, *p* = 0.264. When the reference point was the other, matched with feasibility (M = 6.446, SD = 0.989), the user’s environmental maintenance attitude was weaker than when matching with desirability (M = 6.070, SD = 1.033), and there was a significant difference between the two, *p* = 0.034 (see Figure 2).

(b) The pairwise comparison data showed that when the reference point was the self, matched with feasibility (M = 6.472, SD = 0.710), the user’s environmental responsibility perception was stronger than when matching desirability (M = 6.281, SD = 0.936), and there was no significant difference between the two, *p* = 0.296. When the reference point was others, matched with feasibility (M = 6.598, SD = 0.703), the environmental responsibility perception was stronger than when matching desirability (M = 6.161, SD = 1.296), and there was a significant difference between the two, *p* = 0.014 (see Figure 3).

(c) The pairwise comparison data showed that when the reference point was the self, matched with feasibility (M = 6.377, SD = 0.761), the user’s pro-environmental behavioral intentions were stronger than when matching desirability (M = 5.974, SD = 1.241), and there was a significant difference between the two, *p* = 0.05. When the image reference point was others, matched with feasibility (M = 6.226, SD = 1.274), the user’s pro-environmental behavior intention was weaker than when matching desirability (M = 6.226, SD = 1.274), and there was no significant difference between the two, *p* = 0.285 (see Figure 4).

### 4.3. Conditional Process Analysis

**Figure 5:**
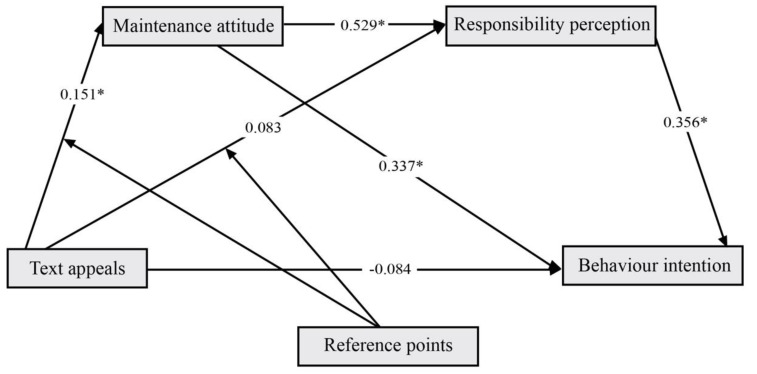
The research framework and path coefficients (the standardized regression coefficient). * *p* ≤ 0.05.

To test the mediating effect of environmental maintenance attitudes and environmental responsibility perceptions, we used PROCESS model 84 with 5000 samples, and the confidence interval was 95%. The model was coded as an image reference point (self reference point = 0; others reference point = 1) and text appeals (feasibility = 0; desirability = 1), and was used to test two independent variables and three relationships between the dependent variables (see Figure 5). The results showed that:

(a) For environmental maintenance attitudes, image reference points and text appeals had an interactive effect on attitudes, and image reference points moderated text appeals (β = 0.151, BootLLCI = 0.023 to BootULCI = 0.278);
(b) For environmental responsibility perception, environmental maintenance attitude had a mediating effect (β = 0.529, BootLLCI = 0.325 to BootULCI = 0.755);
(c) For pro-environmental behavior intentions, both environmental maintenance attitudes and environmental responsibility perceptions had a mediating effect (β = 0.337, BootLLCI = 0.179 to BootULCI = 0.548), (β = 0.356, BootLLCI = 0.179 to BootULCI = 0.523). The data analysis results showed that H2, H3 are correct. There are two paths for the realization of pro-environmental behavior intentions. In the first pathway, mediated by environmental maintenance attitudes alone, image reference points moderated the effects of text appeals on environmental maintenance attitudes (β = 0.051, BootLLCI = 0.152 to BootULCI = 0.214), and then influenced pro-environmental behavioral intentions. The second pathway was mediated by environmental maintenance attitudes and environmental responsibility perceptions. Here, image reference points moderated text appeals of environmental maintenance attitudes (β = 0.028, BootLLCI = 0.007 to BootULCI = 0.131), which affected environmental responsibility and perceived pro-environmental behavior intentions.

## 5. Discussion

This study examined the effectiveness of constructing tailoring relationships between image and text appeals in promoting public environment maintenance behaviors. When the information reference point and the text appeals show a matching relationship, this can encourage the maintenance behavior intentions of the public. The results showed that: (1) image reference points and text appeals have no direct effects on environmental maintenance attitudes, environmental responsibility perceptions, or pro-environmental behavior intentions, but have interactive effects; (2) when the image and text match, the information construction method of combing the other’s image reference point with the desirability of text appeal optimally promotes pro-environmental behavioral intention; and (3) promoting pro-environmental behavioral intentions, environmental maintenance attitudes, and environmental responsibility perceptions all have meditating effects. There are two ways to realize this effect: The first path is an image reference point that moderates the effects of text appeals on environmental maintenance attitudes and promotes pro-environmental behavioral intentions. The second path involves using the image reference point to moderate the text appeals to the environmental maintenance attitude, and environmental maintenance attitude and environmental responsibility perception mediate the effect of environmental maintenance attitude on pro-environmental behavior intention. Environmental responsibility perception does not play a direct mediating role.

In previous research on information dissemination, scholars have been more inclined to explore the construction of text descriptions. Our experimental study innovatively integrates the image perspective information reference point and tries to establish the information matching relationship of text appeals to promote pro-environmental behavior construction. This is not only more in line with the user’s capacity for the fast cognition of information in an era of reading pictures, but it also enriches the interesting nature and beauty of the information on the screen. The results show that image reference points and text appeals have no main effects on environmental maintenance attitudes, environmental responsibility perceptions, and pro-environmental behavioral intentions. In other words: (a) when the text appeals combined with others’ image reference point or self’ image reference point in feasibility appeal, or with others’ image reference point or self’ image reference point in desirability appeal, there were no differences in environmental maintenance attitude, environmental responsibility perception, and pro-environmental behavioral intentions; and (b) when the image reference points combined with feasibility appeal or desirability appeal on the others’ image reference point, or with feasibility appeal or desirability appeal on the self’ image reference point, there were no differences in environmental maintenance attitude, environmental responsibility perception, and pro-environmental behavioral intentions. That is to say, neither the text appeals nor the image reference point can produce a single-factor significant difference on environmental maintenance attitude, environmental responsibility perception and pro-environmental behavior intention. This result has the same conclusion as previous related research on the construction of text information matching. When it is combined with other factors, such as risk tasks, temporal framing, unethical behavior etc. [78,79,80]. It may play a role in the influence of perception, attitude, and behavioral intention regulation.

Although few studies have addressed the interaction of image reference points and textual appeals, our study found that image reference points and text appeals had interactions in the environmental maintenance attitudes, environmental responsibility perceptions, and pro-environmental behavioral intentions three dependent variables. Our study is highly consistent with related research on behavioral intentions in communication and social psychology [81,82,83]. First, according to the construal level theory, “self” has a closer and more specific perception of psychological distance information, while “others” has a farther and more abstract perception of psychological distance information [84]. In the information interpretability appeal, feasibility has a more specific information appeal, while desirability has a more abstract information appeal [85]. There is a strong correlation between the psychological distance dimensions and the construal level [86], therefore, when combined information presents matching patterns, it is more effective than using non-matching patterns in the information construction process. That is, the combination form one: the others‘ image reference point + desirability text appeal and the combination form two: the self image reference point + feasibility text appeal are better than the combination form three: the others‘ image reference point + desirability text appeal and the combination form four: the self image reference point + feasibility textual appeal information combination. This is in line with the social psychology middle school’s action recognition theory [87], and the attitude representation theory [88]. Compared with the “why”-level approach to future behavior in the combination of the first perspective and concrete explanatory text, the use of a third person perspective and abstract explanatory text approaches the “how” level of future behaviors. Identifying high-level behaviors promotes and enhances the perceptions of and attitudes towards future environmental maintenance behaviors and establishes a broader relationship with future environmental protection behaviors. To achieve environmental maintenance behaviors, we must promote reinforcement.

Environmental maintenance attitudes mediate pro-environmental behavior, which is consistent with multiple interpretations of the internal attribution of behavioral intentions in environmental communication. That is, attitude is the strongest factor in the influence of pro-environmental behavioral intentions, and pro-environmental behavioral intentions have an important impact on environmental protection behaviors [89,90]. Therefore, the formation of attitudes at the level of internal attribution is crucial to assuring civilized behavior during public dining. We are also surprised that environmental responsibility perception at the level of social morality cannot directly influence the intention towards pro-environmental behavior and must be mediated by attitude first. This also shows that, compared with social responsibility, spontaneous subjective attitude is more important, and attitude is a prerequisite for intention formation.

### 5.1. Theoretical Implications

Relevant information adjudication has been widely studied in promoting the formation of users’ behavioral attitudes and intentions, mainly focusing on establishing users’ intentions for healthy behaviors, social event risk perception, green advertising, and other multidisciplinary fields. However, current research on the information construction impact of maintenance intentions in public dining spaces is still in its infancy. This study innovatively incorporates image reference points in information construction and applies them to the field of public health. Through experimental studies, we attempted to explore optimal or sub-optimal matching patterns combined with text appeals and image reference points. The results showed that the information combination of others’ image reference point + desirability text appeal and the self image reference point + feasibility text appeal can be significantly better than the other two combinations, thus effectively affecting diners’ attitudes and potentially influencing intentions to maintain the environment.

### 5.2. Practical Implications

According to the experimental research results, we suggest that in the related image-text combined public welfare propaganda design for public dining environment maintenance, the designer should first choose the information combination design method of others’ image reference point + desirability text appeal for screen presentation, and sub-optimally choose the information combination design method of the self image reference point + feasibility text appeal.

### 5.3. Limitations and Directions for Future Research

Our research still has some deficiencies and needs to be enriched and improved. First, we only constructed combined images and texts to transmit information. Future research can better incorporate more information construction methods and perspectives to enhance pro-environmental behavior intention promotion.

In the construction method, we included multimodal fusion designs such as auditory information and dynamic information. In the construction of angles, we included angles that are more similar to the gender or role of the audience in an attempt to enhance the sense of substitution and immersive perception.

Second, we only measured four indicators as the dependent variable and mediating measures, and we only approached this from one level of internal attribution and one level of social ethics. More levels or dimensions of internal factors should be used to explore the impacts on environmental maintenance behavior, such as guilt [91], personal regulatory focus [92], behavioral moral judgment [93], environmental hygiene literacy [94], social norms [95], cultural differences [96], and many other factors, which should be explored in a single or mixed mode.

Third, most of the subjects we selected were young and middle-aged diners, and most of them had higher education levels. Different cultural backgrounds, different groups of diners, and different education levels may influence the cognition and interpretation of information semantics. Therefore, more extensive coverage and multi-dimensional analysis are needed in future research.

In the end, our research stops at the effect of audiences on pro-environmental behavioral intentions. Substantive interventions for future environmental maintenance behaviors and quantitative measures of behavioral effectiveness still require extensive work.

## 6. Conclusions

This research innovatively explored the construction method of image information tailoring to promote public environment maintenance behavior intention. We found that pro-environmental maintenance behavioral intentions were amplified when image reference points matched the textual construal level. The optimal design method combines the use of the other’s image reference point with textual desirability. The sub-optimal design method uses the self’s image reference point combined with textual feasibility. This design method can be effectively applied to E-posters, E-advertisements, or public welfare publicity in various interfaces to the end of improving public dining environment maintenance, and to establish and promote the audience’s intention to carry out environmental protection behavior. Our intention is that every diner will have a clean and tidy dining environment.

## Figures and Tables

**Table 1:** Effects on maintenance attitudes, responsibility, and behavioral intention.

Sources of Variation		Maintenance Attitude		Responsibility Perception		Behavior Intention	
		F	η2	F	η2	F	η2
Image reference points	(1224)	0.001	0.000	0.001	0.000	1.207	0.005
Text appeals	(1224)	0.454	0.002	0.940	0.004	0.456	0.002
Image reference points × Text appeals	(1224)	5.216 *	0.023	6.125 *	0.021	4.686 *	0.020

* *p* ≤ 0.05.

## Data Availability

The data are contained within the article.
